# Generation of epitope-specific hCG aptamers through a novel targeted selection approach

**DOI:** 10.1371/journal.pone.0295673

**Published:** 2024-02-23

**Authors:** Lauren Ferreira, Shane Patrick Flanagan, Ronen Fogel, Janice Leigh Limson

**Affiliations:** Biotechnology Innovation Centre, Rhodes University, Grahamstown, Eastern Cape, South Africa; Danmarks Tekniske Universitet, DENMARK

## Abstract

Human chorionic gonadotropin (hCG) is a glycoprotein hormone used as a biomarker for several medical conditions, including pregnancy, trophoblastic and nontrophoblastic cancers. Most commercial hCG tests rely on a combination of antibodies, one of which is usually specific to the C-terminal peptide of the β-subunit. However, cleavage of this region in many hCG degradation variants prevents rapid diagnostic tests from quantifying all hCG variants in serum and urine samples. An epitope contained within the core fragment, β1, represents an under-researched opportunity for developing immunoassays specific to most variants of hCG. In the study described here, we report on a SELEX procedure tailored towards the identification of two pools of aptamers, one specific to the β-subunit of hCG and another to the β1 epitope within it. The described SELEX procedure utilized antibody-blocked targets, which is an underutilized strategy to exert negative selection pressure and in turn direct aptamer enrichment to a specific epitope. We report on the first aptamers, designated as R4_64 and R6_5, each capable of recognising two distinct sites of the hCG molecule—the β-subunit and the (presumably) β_1_-epitope, respectively. This study therefore presents a new SELEX approach and the generation of novel aptamer sequences that display potential hCG-specific biorecognition.

## 1. Introduction

Human chorionic gonadotropin (hCG) is a glycoprotein hormone secreted by placental trophoblast cells during pregnancy. As such, it is routinely used as a biomarker to diagnose pregnancy in serum and urine samples. hCG is also recognised as a biomarker for: trophoblastic cancers e.g. [1] and other gestational trophoblastic diseases [2, 3], and as a tumour marker for certain other cancers [2, 4, 5].

Research into hCG has revealed a complex biological role and substantial variation in the composition of this hormone. Structurally, intact hCG is a heterodimer consisting of α and β subunits [2, 6]. The α-subunit is identical to that of other glycoprotein hormones: leutenising hormone (LH), follicle-stimulating hormone (FSH) and thyroid-stimulating hormone (TSH) [7]. The β-subunit, although similar to that of LH, is structurally- and functionally-unique and dictates the specific biological activity of the hormone; in hCG, this is largely similar to LH with an additional C-terminus peptide region [7, 8]. hCG present in blood and urine samples encompasses both the natively-expressed, heterodimeric form of this molecule and 14 distinct variants: undissociated β subunits, nicks, truncations, variable glycosylation isoforms, and combinations thereof [2, 7, 9, 10]. A region of the β-subunit, the β core fragment, is conserved across all hCG heterodimer and β-subunit variants [7, 11].

The primary biological function of hCG is the replacement of luteinising hormone (LH) in stimulating and maintaining progesterone production during the early stages of pregnancy [7, 9, 12]. Changes to both the levels of total hCG (rapidly increasing in the first trimester and slowly decreasing thereafter) and the proportions of circulating hCG variants occur as pregnancy advances [2]): from predominantly hyperglycosylated, undissociated, variants being secreted by the developing foetus in the initial weeks following implantation [4, 12] to mostly intact, dimeric, hCG hormone circulating in the second and third trimesters [9]. The levels and types of hCG are both of diagnostic relevance: abnormally high or low levels of hCG or its variants can indicate adverse pregnancy outcomes [2, 7, 13].

There is, thus, a need to develop rapid diagnostic tests capable of recognising and quantifying all hCG variants in both serum and urine samples [11, 14]. Most of the currently available methods to detect and monitor hCG levels are antibody-based, sandwiching, immunoassays: one epitope-specific antibody captures hCG, while a second antibody, specific to another epitope, reports on target presence [15]. Many commercial hCG tests rely on a combination of antibodies specific to the hCG-specific C-terminal peptide (CTP) of the β-subunit and two epitopes present on the core fragment of the β-subunit–β_2_ and β_8_ [7, 14, 16]. The heterogeneity of hCG variants in human samples complicates the accurate measurement of this analyte by this approach, as various epitopes are either missing or masked (e.g. by glycosylation state) in specific hCG variants. This, leads to significant inaccuracies in hCG detection and quantification using a paired antibody approach, both in lateral flow assays e.g. [17] and in clinical assays e.g. [11].

In contrast to existing detection platforms, a diagnostically-useful epitope contained within the core fragment, β1, represents an under-researched opportunity for developing specific immunoassays for hCG [16]. Biorecognition elements specific to this epitope are reported to offer superior specificity, a wide recognition of hCG and its variants, and the absence of cross- reactivity with LH [16]. In addition to the above, limitations in conventional hCG-detecting immunoassays, antibody-based diagnostic tools provide high target specificity and affinity but have high production costs and limited shelf lives, due to their thermosensitivity [18].

DNA- or RNA-based aptamers boast several advantages over antibodies, and a number of strategies have been described to obtain epitope-specific aptamers [18–21]. In the study described here, we report on a SELEX procedure tailored towards the identification of two pools of aptamers, one specific to the β-subunit in general and another to the β1 epitope of hCG. This was achieved using a novel selection approach to enrich sequences binding to distinct regions of the target, within a single SELEX experiment.

## 2. Materials and methods

### 2.1 Reagents

#### 2.1.1 Oligonucleotides for SELEX and binding studies

Oligonucleotides in this study were sourced from Integrated DNA Technologies (WhiteSci). For SELEX, a library of the general sequence: 5′-TCGCACATTCCGCTTCTACC(N_40_)CGTAAGTCCGTGTGTGCGAA-3′ [22–25] was used. The randomised regions were prepared combinatorially by mixing A : C : G : T nucleotide bases at molar ratios of 3 : 3 : 2: 2.4, to optimise equal probability of incorporation of each nucleotide [25].

Two sets of oligonucleotides were used with the library in this study. The first, a set of primer pairs (Forward: 5′-TCGCACATTCCGCTTCTACC-3′ and Reverse: 5′-TTCGCACACACGGACTTACG-3′) was used to amplify the aptamer candidates between rounds of SELEX; the reverse primer was sourced with a 5′-phosphorylation modification, to allow production of single-stranded DNA by exonuclease digestion. The second set of synthesised oligonucleotides (Forward: 5′-GGTAGAAGCGGAATGTGCGA-3′ and Reverse: 5′-TTCGCACACACGGACTTACG-3′) were designed to complement primer binding sites on the library and was used during SELEX to hybridise to these regions.

#### 2.1.2 Proteins and antibodies

hCG (≥50%, isolated from human urine, ab126652) was purchased from Abcam; Follicle-Stimulating Hormone (FSH) (7000 I.U./mg, F4021) and human serum albumin (HSA) (99%, A3782) were purchased from Sigma-Aldrich. All proteins were received as lyophilised powders and resuspended in 0.15 M HEPES, adjusted to pH 7.0, as stocks: 1 mg/ml (hCG), 0.25 mg/ml (FSH) and 0.25 mg/ml (HSA), respectively. Aliquots of each protein were made and stocks were stored at -20°C until used.

Monoclonal mouse anti-hCG beta1 epitope antibody (1 mg/ml, ab11388, Abcam), hereafter referred to as the hCG β_1_-epitope specific antibody, was used as a primary antibody to hCG. Polyclonal goat anti-mouse IgG, conjugated with horseradish peroxidase (HRP) (0.1 mg/ml, HAF007, R&D Systems) was sourced as a secondary antibody. Polyclonal goat anti-hCG alpha antibody (2.11 mg/ml, ab20712, Abcam), hereafter referred to as the α-subunit-specific antibody, was used as a primary antibody binding to hCG and FSH. Polyclonal rabbit anti-goat IgG, conjugated with HRP (2 mg/ml, ab6741, Abcam), was sourced as a secondary antibody to the α-subunit antibody. HRP presence was detected using 1-Step UltraTMB-ELISA (ThermoFisher Scientific) for magnetic bead assays, or using the KPL TMB membrane peroxidase substrate system kit (SeraCare), where applicable.

DynaBead™ epoxy-functionalised M-270 superparamagnetic beads (ThermoFisher Scientific) were used as a solid support for protein coupling in the SELEX experiment.

### 2.2 Methodology

#### 2.2.1 SELEX

SELEX proceeded by incubating ssDNA library pools with magnetic beads surfaced with the target proteins. Exposure of the DNA pools to the proteins occurred in a synthetic urine matrix at 21 ± 2°C consisting of: 200 mM urea, 1 mM uric acid, 3.43 mM creatine, 0.1 mM sodium oxalate, 5 mM sodium citrate, 54.2 mM sodium chloride, 30.2 mM potassium chloride, 15 mM ammonium chloride, 3 mM calcium chloride, 2 mM magnesium sulphate, 2 mM sodium bicarbonate, 9.1 mM sodium sulphate, 3.6 mM sodium dihydrogen phosphate, and 0.4 mM disodium hydrogen phosphate; pH 6.2 [26]. The urine matrix was supplemented with 2.8 nM glucose and 17 nM HSA and was freshly prepared before each selection round. After incubation with the beads, bead-binding or non-binding fractions of ssDNA were isolated and selectively amplified for the next round of selection.

**Fig 1:**
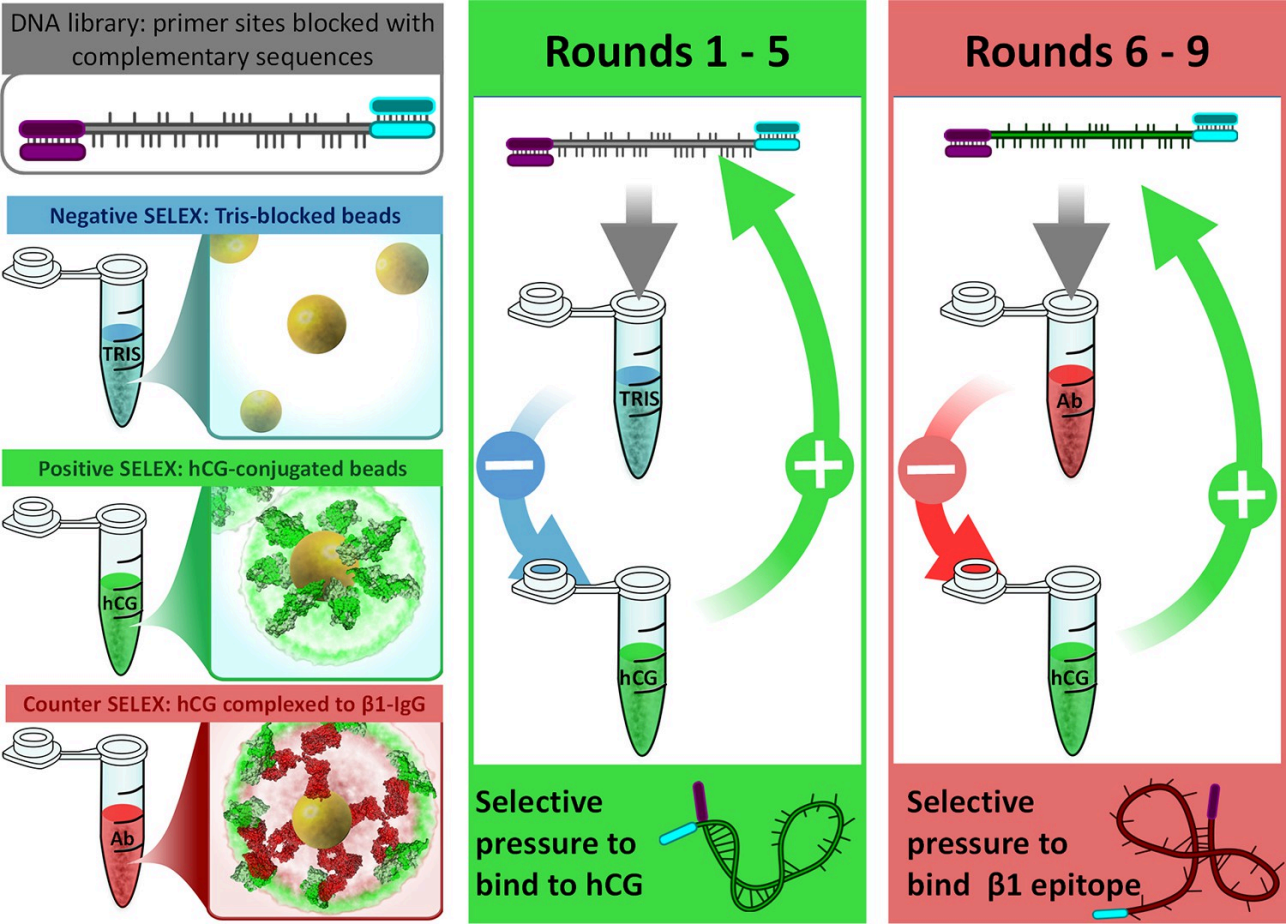
Selection strategy used during SELEX to enrich for multiple, site-specific DNA aptamers.

Selection occurred in two phases in an attempt to identify aptamers capable of binding to the β-subunit as a whole and also the β_1_ epitope specifically (Fig 1). Further details of the various counter/negative and positive selection steps used, as well as the stringency applied, are summarised in S1 Table in S1 File. Using this strategy, negative, counter, and positive selection phases were determined by controlling the protein modification present on the magnetic beads. After either a negative or counter selection phase, the unbound sequences remaining in the supernatant were used in the second, positive selection step. All rounds of SELEX employed the same positive selection pressure—hCG-functionalised magnetic beads.

Two stages of selection were used to enrich aptamers capable of recognising distinct sites of hCG. Representations of hCG and IgG are drawn from the RCSB Protein DataBase; PDB accession codes: 1HRP.PDB [6] and 1IGY.PDB [27], respectively.

In the first stage of SELEX (Fig 1, “Rounds 1–5”), negative selection occurred against Tris-blocked beads, thereby removing sequences recognising the solid support of the magnetic beads. Positive selection was against beads modified with the target protein, hCG. For the second stage (Fig 1, “Rounds 6–9”), negative selection pressure changed to a counter-selection, using beads containing the β_1_-epitope specific antibody complexed to hCG. Any DNA sequence binding to the remaining exposed sections of the hCG molecule would bind to this bead and be removed from the pool; following positive selection, only sequences specific to the β_1_ epitope would be enriched. Within each of the stages (Rounds 3 and 7), an additional counter selection was performed, using beads modified with FSH as a negative selection pressure, in order to remove aptamers capable of binding to the alpha subunit shared between FSH and hCG.

Magnetic beads were coupled to the relevant protein (S1 Table in S1 File) according to the manufacturer′s instructions. After protein incubation, any residual excess protein was removed by collecting the beads using a magnetic separator and aspirating the supernatant. The beads were washed four times with 1 ml 0.15 M HEPES, pH 7.0. Beads were blocked with 0.2 M Tris, pH 8.5 for one hour at 4°C, washed four times with HEPES then used immediately for library pool exposure. For rounds 6 to 9, hCG was incubated with the antibody-coated beads after Tris blocking and allowed to bind for two hours at 21 ± 2°C, with gentle rotation, before being washed and used.

The beads were resuspended in 1 ml of artificial urine matrix containing the folded, blocked aptamer library pool and incubated at 21 ± 2°C with gentle rotation (see S1 Table in S1 File for DNA quantities and incubation times). To ensure annealing of the complementary oligonucleotides and proper folding of the variable region of each library member, equimolar amounts of library and the two blocking oligonucleotides were mixed in an artificial urine matrix. This solution was heated to 95°C for five minutes then slowly cooled to 21 ± 2°C at a rate of 0.5°C per minute in a 3Prime thermocycler (Techne), before incubation with functionalised magnetic beads.

During negative selection steps, the beads were collected by magnetic separation and the supernatant containing aptamer candidates was immediately transferred to the prepared positive selection beads. During positive selection, the ssDNA pool remaining in the supernatant was allowed to bind to the target beads at 21 ± 2°C for between 45–60 minutes, depending on the round (S1 Table in S1 File). During the counter selections occurring at rounds 3 and 7, sequences still suspended in the supernatant after FSH binding were collected and precipitated. For all positive selection rounds, the supernatant after target binding was removed and any non-specifically or weakly bound sequences remaining on the beads were washed off with increasingly-stringent regimens as SELEX proceeded (S1 Table in S1 File).

Bound DNA sequences remaining on the target beads were eluted with the addition of 200 μl of elution buffer (40 mM Tris, 10 mM EDTA, 3.5 M Urea, 0.002% TWEEN® 20, pH 8.80), heating to 80°C for ten minutes each [28]. After collection of the supernatant, elution from the beads was repeated and the pools combined. Eluted ssDNA was recovered by precipitation: firstly sequentially adding and mixing 3M sodium acetate (pH 5.5) and glycogen to the combined eluants (to final concentrations of 0.225M and 0.165 g/L, respectively); adding 1 ml of ice-cold 96% ethanol to the mixtures and incubating this overnight at -20°C; collecting the pellet by centrifugation (30 minutes at 12,000×g at 4°C); washing the residual pellet using 1ml of 70% ethanol and recentrifuging the pellet; and finally air-drying the pelleted DNA. Once dry, this was resuspended in 30 μl H_2_O [29].

Portions of the recovered DNA pool were briefly amplified by PCR for two cycles to generate dsDNA, for improved stability. The ssDNA was amplified using KAPA Taq (Sigma), with 0.25 μM of each primer, using the phosphorylated reverse primer.

To generate sufficient copies of the recovered sequences for use in the following SELEX round, the DNA was further amplified using the same conditions. Amplification was stopped at an optimal number of amplification cycles (i.e. the number of PCR cycles generating maximal product but before higher molecular weight amplification artefacts were visible on a 1×GelRed (Biotium) stained 10% PAGE gel). The ssDNA pool for the next round of selection was regenerated from the amplified product using 50 U lambda exonuclease (New England Biosciences) at 37°C for one hour, similarly to previously described [25, 30, 31].

#### 2.2.2 Quantitative real-time PCR

ssDNA eluted from both positive and negative selection beads, at each SELEX round, was directly quantified by qPCR using Quantinova SYBR Green Master Mix (Qiagen) and 3.5 μM of each primer. Real-time amplification was performed in a CFX Connect™ Real-Time PCR Detection System (BioRad). The thermal cycling conditions used were: initial denaturation at 95°C for 2 minutes, then 40 cycles each of denaturation at 95°C for 5 seconds, annealing at 64°C for 20 seconds and extension at 72°C for 10 seconds.

The DNA pool diversity of each selection round was also assessed by examining the shape of the amplification plots and melt curves obtained from the qPCR quantification of the exonuclease digested samples from each round. Melt curve analysis was performed by cooling the amplicons to 55°C after the final extension step, then gradually heating to 95°C and monitoring fluorescent intensity. C_q_ values obtained from the CFX Manager Software were used to construct standard curves based on diluted library samples of known concentration, included on every individual plate.

The binding affinity of the ssDNA pool at various SELEX rounds was estimated by quantifying the amount of DNA bound to hCG-functionalised beads directly. Briefly, 1 pmol of exonuclease digested ssDNA, with equimolar amounts of the complementary blocking oligonucleotides, was heat-denatured and cooled back to 21 ± 2°C. The complemented and folded DNA was introduced to 1 mg of magnetic beads functionalised with 10 μg of hCG. After binding for one hour at 21 ± 2°C with gentle rotation, the beads were collected and the supernatant containing any unbound DNA sequences was removed. After resuspending the beads in 1 ml fresh artificial urine matrix, 1 μl of the resuspended beads were used directly as the template in qPCR for DNA quantification. This was compared to the quantification of the original input DNA and the unbound fraction in the supernatant.

#### 2.2.3 NGS library generation, sequencing and analysis

Next-Generation Sequence (NGS) libraries for MiSeq sequencing (Illumina) were generated from recovered pools from various SELEX rounds (1, 4, 5, 6, 8 and 9), as well as the initial, unselected library. A no-template control (NTC) pool was created by PCR amplification of a sample containing the forward and reverse primers, but lacking any template, for 30 amplification cycles using the same methodology and thermal programme outlined for PCR in Section 2.2.2.

Illumina-specific primers were ligated onto each sample pool by reacting a 1 μl aliquot of each pool with 0.25 μM of each Illumina-specific primer (F: 5′-TCGTCGGCAGCGTCAGATGTGTATAAGAGACAGTCGCACATT CCGCTTCTACC-3′ and R: 5′-GTCTCGTGGGCTCGGAGATGTGTATAAGAGACAGTTCGCACACACGGACTTACG-3′; underlined nucleotides indicating the constant primer binding regions of the SELEX pool) for 6 amplification cycles. Following PCR, the desired amplicon was purified via gel excision after electrophoresis within a 2.5% ^w^/_v_ agarose gel. The purified 147 bp product was submitted to the NRF-SAIAB Aquatic Genomics Research Platform (Makhanda, South Africa) for Illumina library generation and paired-end 75 bp MiSeq sequencing.

Samples were multiplexed with unique barcodes for each pool and sequenced together on a single MiSeq flow cell. Paired-end FASTQ datafiles were demultiplexed, based on the barcodes. Sequences from each sample were extracted and analysed separately using AptaSUITE [32]. Sequences were then clustered using the AptaCLUSTER package [33] contained within the AptaSUITE collection, using an edit distance of 2 substitutions/insertions/deletions to cluster sequences. Subsequently, clusters were collected as central sequences, ranked in order of frequency of occurrence within each pool within a text file, for further analysis. The most-abundant 15,000 clusters in each pool were compared with one-another in order to assign their emergence, enrichment and removal during the different phases of SELEX.

The frequency-ranked lists of unique sequences for each round were assigned a name to each sequence, depending on which round they initially appeared in, and their position in the ranked list (for example, R4_1 was the most abundant sequence first identified during the fourth round of SELEX). Candidate aptamers were chosen based on their abundance and enrichment through selection and their response to changing selection parameters, relative to control library pools.

#### 2.2.4 Screening of candidate aptamers

Aptamer candidate sequences identified through SELEX in these studies, as well as control sequences were sourced from IDT for empirical testing in validation studies. Table 1 presents their sequences and designations:

**Table 1:** Aptamer sequences and their variations used in this study.

Name	Variable length region sequence (5′ → 3′)	Variations
Lib_1	TATGCCCAAATCCCTCAAGTCGGCCAGGATACACCAC	Full-length sequence i.e. including primer-binding sites.
R4_1	TGGACGAGGTATCTCGTATCATTGGCACCTAAAACTCCGT	Full-length. Variable region only, with 5′-biotin
R4_64	ACCTGCTGACATTGGGTGGGTCCTGAACCATTTTTAATCA	Full-length, unmodified or 5′ biotin. Variable region, 5′-biotin or 3′-biotin.
R5_4	CAAGGATCGGCTCAACTTAATATTGGAGGGATAGTGTTGG	Full length. Variable region, 5′-biotin.
R6_5	CAACTATTTCAGTATTCTAGAAAGGTCAAACCTTAGGAGC	Full length. Variable region, 5′-biotin.

Both truncated sequences (i.e. sequences comprising only the variable regions of the aptamer candidates) and full-length sequences were sourced as required (Table 1). Truncated sequences, modified on the 5′ end with a biotin moiety, were used to initially screen target-binding ability by ELONA methods. Other studies e.g. screening of aptamer candidates’ binding by qPCR using the full-length DNA sequence was also conducted.

S3 in S3 File the Supporting Information provides detailed methodology used to screen the aptamer candidates. Selected sequences were compared to one-another: via electrophoretic migration shift assays with both truncated aptamer and hCG dissolved in solution (S3.1 in S3 File); via magnetic bead Enzyme-Linked OligoNucleotide Assays (ELONAs), immobilizing hCG onto the surfaces of microtiter plate wells and comparing binding of biotinylated truncated sequences using streptavidin-HRP enzyme activity (S3.2 in S3 File); via magnetic bead capture ELONAs, immobilizing biotinylated full-length aptamers onto streptavidin-modified magnetic beads and estimating the amount of hCG retained by those beads via binding of the α-subunit antibody using an HRP-conjugated secondary antibody (S3.3 in S3 File); via paper-based ELONAs, immobilizing hCG onto cellulose surfaces via EDC/NHS coupling and estimating binding capabilities of biotinylated truncated aptamers to those surfaces using streptavidin-HRP enzyme activity (S3.4 in S3 File); and via qPCR estimation, by functionalising magnetic beads with HRP, exposing the beads to full-length aptamer sequences, and estimating the aptamers retained at those beads via qPCR (S3.5 in S3 File).

### 2.3 Statistical analysis

Unless otherwise stated, tests requiring repeated measures were performed in, at least, triplicate samples i.e. n ≥ 3. For univariate plots, means are shown as solid lines, while medians are dashed. Results reported in text represent means ± standard deviation. Statistical differences between two samples were evaluated using two-tailed, equal-variance, Student′s *t*-tests. One-way ANOVA analyses, with Tukey HSD *post hoc* tests, were used for determining and comparing significant differences in the means of more than two samples. Statistical significance (α) was assigned at 0.05.

## 3. Results and discussion

### 3.1 Targeted SELEX strategy produced aptamers specific to different epitopes of hCG

Nine rounds of a novel, SELEX strategy were completed to enrich DNA aptamers capable of binding to distinct sites of the hCG target molecule (Fig 1). This strategy utilized antibodies to mask the β1 epitope of hCG, and removed sequences with affinity to other sites of hCG and the bead-matrix support. Counter selection against the related glycoprotein hormone, FSH, which shares an identical α-subunit complexed to a different β-subunit [7, 12, 34], was also performed to ensure identification of DNA aptamers specific to the β-subunit of hCG.

**Fig 2:**
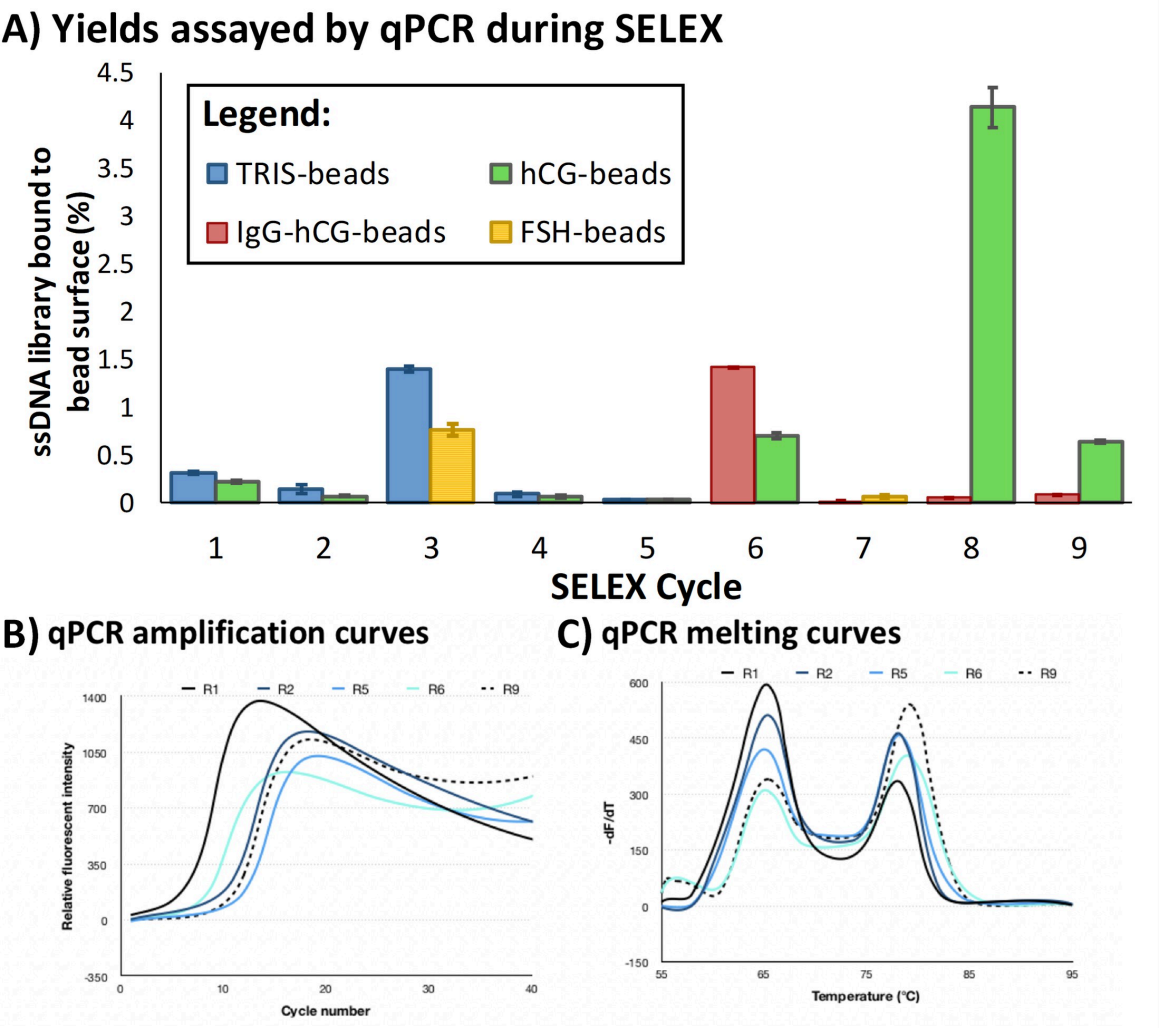
Monitoring of the progress of selection through SELEX by qPCR using samples of ssDNA eluted from the surfaces of beads used during SELEX. **A)** Absolute DNA quantity obtained by qPCR for negative and positive selection steps at each round of selection. **B)** Amplification curves obtained for different selection rounds analysed by qPCR. **C)** Melting curves obtained for different selection rounds analysed by qPCR.

The quantity of DNA (as well as some preliminary analysis of sequence diversity) eluted directly from the magnetic beads, for both positive and negative selection steps at each round, was estimated using qPCR of fractions of the pool (Fig 2A).

Similar to this study, quantification of retained DNA at each selection round is often used as a method to monitor SELEX progress [35]. However, these reports do not generally consider any differences in target availability at individual rounds, nor do they (generally) report on quantities other than those obtained from positive selection stages.

With the enrichment of aptamers within the DNA pool through selection, a concomitant increase in binding affinity for the target, hCG, by the pools was expected as SELEX progressed e.g. [31, 35]. The enrichment of target-binding DNA sequences was monitored through the selection process by quantifying the DNA eluted from the beads during SELEX (Fig 2A). Initially, only a small proportion of the oligonucleotide library bound to the beads modified with the hCG target (0.22 ± 0.02% of the input DNA), initially decreasing as SELEX stringency increased SELEX (to ~0.07% of the initial pool by Round 5, the end of the first stage of SELEX), but increasing to a maximum of 4.13 ± 0.21% by the end of Round 8. Generally, in the first phase of selection (Rounds 1–5), more ssDNA bound to the beads used in the negative selection steps than to the positive selection beads (Fig 2A). However, once the selection pressure was changed from the sixth round, more DNA was eluted from the positive selection beads than those used in the negative selection step, despite the increasing stringency applied through selection (S1 Table in S1 File). These findings show the changing behaviour of the DNA pool through selection and indicated an increased affinity towards the target as SELEX progressed.

In addition to quantification of the recovered DNA pools through selection, qPCR analysis of the exonuclease digested samples from each SELEX round was used to monitor changes in pool diversity by examining the amplification plots (Fig 2B) and melting profiles (Fig 2C) e.g. [24, 35–38]. The qPCR technique provides a rapid method to not only quantify DNA through SELEX but also to estimate pool diversity.

Distinctive decreases in fluorescent intensity after peak fluorescences were reached during qPCR amplification indicate a complex and heterogeneous template [24]. These are evident in qPCR profiles from early selection rounds (e.g. R1 and R2 in Fig 2B); as selection progresses, this decrease becomes less pronounced, indicating convergence of the sequences present in the DNA pool at later rounds of SELEX e.g. R9 [24, 36]. Similarly, the shape and position of the peaks of DNA melting profiles changes as SELEX proceeds (Fig 2C), which also indicated convergence of the DNA pool at later selection rounds. Hetero-duplexed amplicons, characterised by a melt peak centered around 67°C predominate at the earlier selection rounds (e.g. R1, Fig 2C) [25, 36]. Towards the end of selection at round 9, homo-duplexed DNA sequences with defined peaks at higher melt temperatures of ~ 81°C predominate the pool (e.g. R9) [25, 36].

### 3.2 Next-generation sequencing analysis, coupled with the targeted selection strategy, allowed site-specific aptamers to be identified

Samples of seven ssDNA pools from key selection rounds (R1, R4, R5, R6, R8 and R9), in addition to control pools (a sample of the starting library and a DNA pool created in the absence of template), were analysed by next-generation sequencing (NGS) to investigate their sequence composition and evolution through the targeted selection strategy. Individual samples were uniquely barcoded to allow parallel, multiplexed sequencing on a single Illumina MiSeq flow cell. The No-template control pool was imported from a separate SELEX study utilizing the same library.

**Fig 3:**
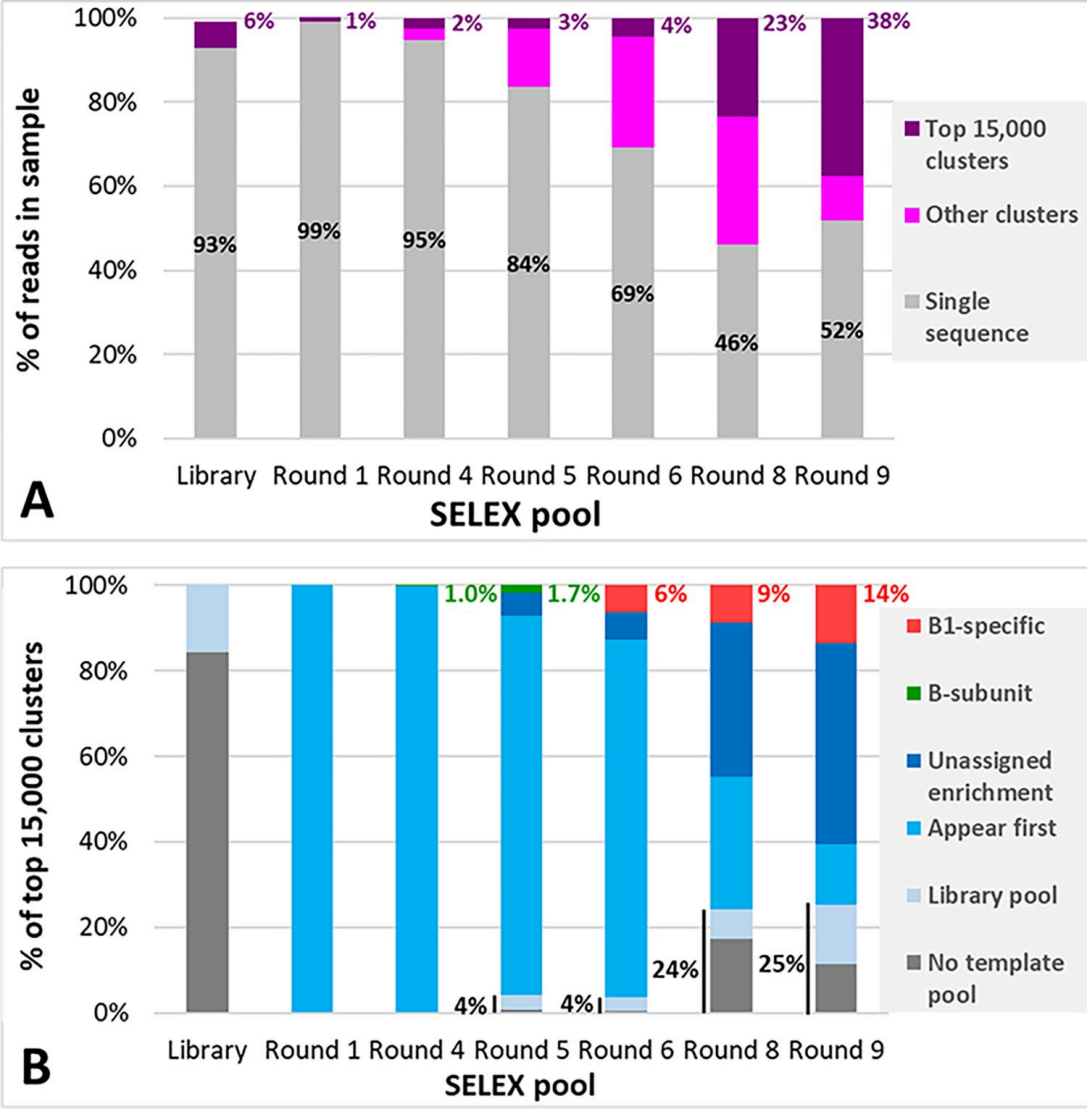
Bioinformatic analysis of sequence enrichment as SELEX progressed, showing the groups of sequences anticipated from the SELEX procedure in this study.

Over 1 million paired-end reads were obtained for each sequenced sample. On average, 94.48% ± 0.36 of reads for each sequenced round passed AptaSUITE quality controls, after demultiplexing the barcodes, merging the paired-end reads and trimming off primer sequences (S2 Table in S2 File). After clustering analysis using Aptacluster, the most-abundant 15,000 families of aptamers were manually compared with one-another to ascertain whether enrichment of specific clusters of sequences was occurring. These results are presented in Fig 3, below.

**3) Cluster analysis of pools**.

Reads from S2 Table in S2 File are presented as the percentage of abundances of: single sequences i.e. n = 1 sequence in sample pool; those identified as clusters i.e. >1 sequences and those sequences corresponding to the top 15,000 most-abundant families of clusters as identified using AptaCluster.

**B) Identification of the categories that the top 15,00 Clusters fall into**.

Sequences were assigned categories based on which samples of the SELEX pool they were first identified in.

Abundance of sequence clusters monitored through SELEX was cibdycted by examining the AptaSUITE cluster size at each sampled round, with expected binding behaviour under changing selection pressure used to identify different categories of potential hCG-specific aptamers.

The number of unique reads present in each sequenced sample steadily decreased through selection (Fig 3A): from between 98.8% (initial library pool) and 99.1% (after one round of selection) to an apparent plateau of approximately 50% unique reads by the end of SELEX (Round 8: 46.10%, and Round 9: 51.76%). Concomitantly, the copy number of individual AptaSUITE seed sequences (i.e. the abundance of specific sequences appearing in the SELEX pool samples) increased through selection. Of the clusters found, an increasing proportion were part of the top 15,000 clusters within each pool. The increase in abundance of specific sequences and specific clusters of sequences demonstrate a decreasing heterogeneity of the pool expected for the enrichment of particular sequences through SELEX and predicted by the initial qPCR characterised on of each pool (Fig 2C and 2D).

The predicted binding characteristics of sequences selected at various rounds of SELEX, could be identified, based on which sample pool they were first identified in (Fig 3B). NGS analysis of the initial, unselected library (“Library Pool” and a library composed of PCR-amplified primers without the presence of template molecules (“No Template Pool”) revealed the presence of some predominant sequences (approximately 6% of the sample) already present within the sample pool. These were found to be mainly derived from primer-based artefacts (based on sequence similarity to the “No Template” pool) and indicated the presence of a similar level of amplification artefacts found throughout further samples findings, similar to other reports e.g. [39]. The remainder of the sequences clustered within the library pool were assigned to a combination of bias within the solid-phase synthesis process resulting in reduced library diversity and complexity, and/or additional amplification artefacts caused by the presence of template during PCR cycling, similar to other findings [39–41]. Combined, clusters of sequences initially found in the library or template-free control pool were at very low concentrations at the beginning of SELEX, but increased significantly as SELEX progressed, becoming 25% of the most-abundant clusters by the end of round 9. The inclusion of both these sample pools during analysis of the clusters allowed subsequent occurrences of these sequences to be excluded as potential aptamers, due to their presence in the control samples.

Four broad groups of binders could be characterised by monitoring the changes in the cluster sizes as selection progressed (Fig 3B). The first group are sequences binding to the β_1_ epitope of hCG (“hCGβ_1_ epitope-specific”)–which were assigned as sequences that became enriched between Rounds 6–9. While only a small proportion of clusters were initially found in the round 6 pool (6% of clusters), this significantly increased as SELEX progressed; at the end of SELEX, approximately 14% of the top 15,000 clusters were considered as β_1_-specific epitopes. The second group–sequences binding to the β subunit of hCG, but definitely not at the β_1_ (“hCGβ-specific”)—were assigned to sequences that would be enriched between rounds 1–5, but begin to decline in frequency following the change in target in round 6. A much smaller proportion of the pool corresponded to this criteria–by the end of Round 5, only 1.7% of the clusters exhibited enrichment behaviour consistent with the criteria.

Non-target specific sequences were also identifiable as SELEX progressed, these were assigned to three different groups: “Appear first”, “Unassigned enrichment” and a “No template pool”. The presence of clusters that were unique to the round i.e. appeared only within that round and did not experience further enrichment (”Appear first” samples in Fig 3B) gradually decreased from being the vast majority of the pool in after one round of selection 1 (99.95%) to lower levels by the end of the first selection phase (88.73% by Round 5), becoming a minor fraction of the sequenced pool by the end of SELEX (13.9% by Round 9). This is further evidence of sequence enrichment during SELEX. Additionally, sequences showing sustained increasing enrichment throughout selection—irrespective of the change in target (“unassigned enrichment”)–were considered to be sequences that bound to matrix components that were shared between the rounds (such as the magnetic bead surfaces) or to preferential amplification during PCR between SELEX rounds (similar to those sequences identified in the “No Template” control pool). These become a significant proportion of the pool during amplification–being of negligible abundance in earlier rounds of SELEX to comprising 5.4% of the pool by the end of round 5 and increasing to 47.1% by the end of round 9.

The change in target selection during SELEX between rounds 1–5 and 6–9 allowed for a greater degree in confidence in assigning binding behaviour to the pool, enabling selection of sequences for further analysis that had a higher possibility of selective binding to the identified epitopes. Without comparison to the starting library, and monitoring the change in sequence abundance under changing selection pressure, these sequences would have been indistinguishable from highly enriched and abundant candidate aptamers. Therefore, altering the selection pressure midway through SELEX proved to be a useful and informative method for identifying amplification artefacts which would otherwise have steadily increased in prevalence through selection.

### 3.3 Selected novel aptamers are capable of binding to hCG

Of the most abundant and most highly enriched sequences in each NGS predicted binding category, five sequences (a suspected amplification artefact, R4_1; two β-specific aptamers R4_64 and R5_4 and one β_1_-specific aptamer R6_5) were selected and empirically tested for target binding ability. The most-abundant sequence initially found in both the library and no-template control pools during NGS analysis, Lib_1, served as a negative control.

**Fig 4:**
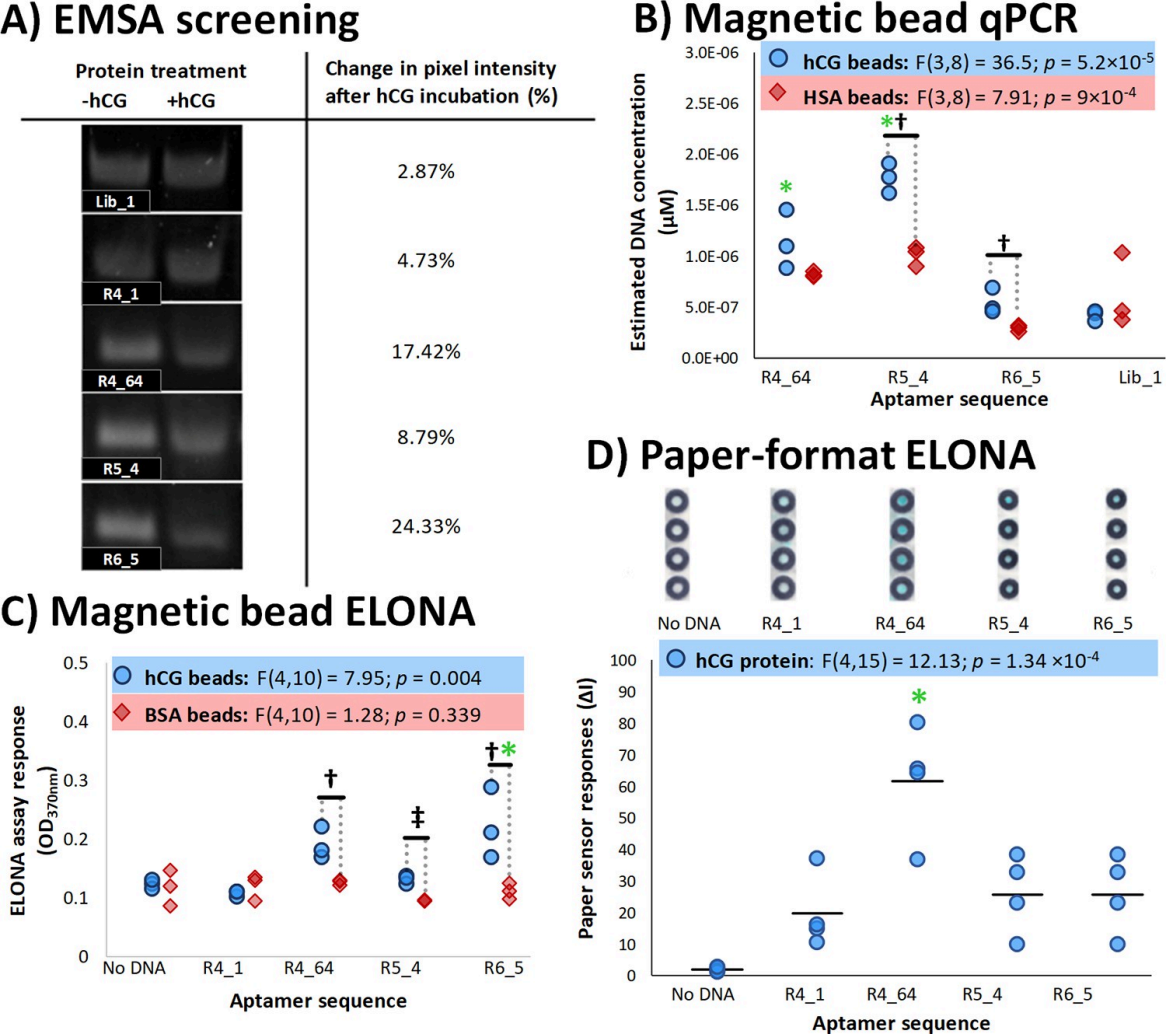
Screening of the ability of selected aptamer sequences to bind to the hCG target, evaluated using a variety of binding assays. **A) Initial screening of aptamer candidates′ ability to bind unimmobilised hCG assessed by EMSA.** Representative GelRed stained gel images are shown with or without hCG incubation. The difference in pixel intensity of the DNA bands for each sequence was calculated in the presence and absence of hCG using ImageJ analysis software. Annotations: **†**- changes in band intensity in the presence of the target ≥ 10% are considered significant. **B) qPCR-based quantification of aptamer sequences binding to beads coated either with hCG or the control protein, HSA.** Assays were performed in triplicate, with all three values shown. Annotations: **†**—Sequences exhibiting significantly higher quantities of DNA bound to hCG-modified beads, compared to HSA-modified beads (two-tailed *t*-tests, *p* ≤ 0.05). *—Sequences exhibiting significantly higher binding to hCG-modified beads compared to the Lib_1 control sequence (one-way ANOVA results annotated in Fig; sequences identified using subsequent Tukey HSD *post-hoc* test, *p* ≤ 0.05). C) Spectrophotometric ELONA assay of biotinylated aptamer sequences binding to beads coated either with hCG or the control protein, BSA. Following exposure of the aptamers to hCG- or BSA-coated magnetic beads, bound aptamers were subsequently quantified using the streptavidin-HRP and TMB reporter system. Assays were performed in triplicate, with all three values shown. Annotations: †—Sequences exhibiting significantly higher quantities of DNA bound to hCG-modified beads, compared to HSA-modified beads (two-tailed *t*-tests, *p* ≤ 0.05). ‡—Sequences exhibiting suggestively higher quantities of DNA bound to hCG-modified beads, compared to HSA-modified beads (two-tailed *t*-test, *p* ≤ 0.1). *—Sequences exhibiting significantly higher binding to hCG-modified beads compared to “No DNA” control samples (one-way ANOVA results annotated in Fig; sequences identified using subsequent Tukey HSD *post-hoc* test, *p* ≤ 0.05). **D) hCG Coated Paper-format ELONA screening of aptamer sequences.** hCG was covalently attached to the paper surface within each barrier and exposed to biotinylated sequences. Bound sequences were subsequently quantified using an anti-biotin antibody and subsequent quantification using a secondary antibody-HRP and TMB system. Representative images captured of the colorimetric signal generated for different aptamer sequences tested by indirect paper-based ELONA (top panel). The signal intensity for each sample was quantified by ImageJ and is presented as a univariate plot (bottom panel). Annotations: *—Sequences exhibiting significantly higher binding to hCG-modified wells compared to R4_1 control samples (one-way ANOVA results annotated in Fig; sequences identified using subsequent Tukey HSD *post-hoc* test, *p* ≤ 0.05).

These sequences were initially screened for their individual binding abilities to hCG, (Fig 4). For qPCR screening and EMSAs, full-length aptamer sequences were tested (Table 1, Fig 4A and 4B). ELONA-style assays (Fig 4C and 4D) used 5′-biotinylated aptamer sequences that lacked their primer binding sites (i.e. truncated to encompass only the variable region, Table 1) to report on the quantities of hCG-bound aptamers. These assays involved differing states of the hCG target and screened aptamers, and assay conditions to assess whether screened sequences were capable of consistently binding selectively to the chosen targets.

EMSA (Fig 4A), allowed both the hCG target and the aptamer candidates to bind freely to one-another in solution. This was selected as the first screening approach, as aptamer immobilisation and/or the conjugation of functional agents to the sequence has previously been shown to influence binding performance (e.g. [42, 43]. Target binding was assessed by incubating the target with full-length aptamer sequences to form aptamer-target complexes, subsequently separating the unbound aptamers by electrophoresis. Aptamer-target binding was evaluated by comparing the band intensity of the band associated with unbound DNA from a sample lane containing aptamers in the absence of the target (“aptamer alone” samples in Fig 4A), to the same band in samples incubated with a ten-fold molar excess of hCG (“aptamer and hCG” samples in Fig 4A) before electrophoresis. A decrease in band intensity of at least 10% in the presence of hCG was taken as evidence of significant binding.

From EMSA (Fig 4A) sequences predicted by NGS analysis to be potential amplification artefact sequences, Lib_1 and R4_1, showed very slight decreases in unbound DNA band intensity after hCG addition during EMSA evaluation (Fig 4A), indicating that little of the DNA had formed hCG-aptamer complexes. These results confirm their poor target-binding affinity and suggest their presence in the SELEX pools arose from initial amplification bias [44] rather than target-binding capability. In contrast, R4_64 and R6_5 exhibited visible decreases in the intensity of their DNA bands associated with unbound aptamer; producing decreases of between 17 and 24%, respectively. Sequence R5_4 showed possible, but not strong, binding ability when assessed by this method. EMSA results provided initial confirmation of target binding ability for some of the novel aptamer sequences, which were verified using other conventional aptamer-binding assay techniques.

In a study designed to mimic SELEX conditions, the selected full-length sequences were subsequently screened for their ability to bind to hCG-surfaced magnetic beads, subsequently detecting the bound sequences by qPCR (Fig 4B). Magnetic beads modified with Human Serum Albumin protein (HSA) were used as controls. HSA was selected as a control protein due to its expected presence at high concentrations within urine samples e.g. [45]. To replicate the experimental conditions used during selection, and limit aptamer fold and function to only the variable region, the aptamer candidates were heat denatured and re-annealed in the presence of oligonucleotide blockers.

Binding of the tested sequences to hCG-coated beads was significantly influenced by the sequence used. R4_64 and R5_4 bound significantly more DNA to the target beads than the control Lib_1 sequence (* annotations and annotated ANOVA results, Fig 4B). All tested aptamers besides Lib_1 showed higher affinity for hCG-modified beads, compared to beads modified with HSA; this preferential binding to hCG was significant for aptamers R5_4 and R6_5 (**†** annotations, Fig 4B) but not R4_64 (t(4) = 1.85; *p* = 0.14). None of the tested sequences exhibited significant binding to HSA-modified beads, compared to Lib1.

A variation of the qPCR assay (Fig 4B) was employed using biotinylated aptamer sequences, creating a colourimetric ELONA (Fig 4C). During the colourimetric ELONA study, 5′ biotinylated full-length aptamer sequences were exposed to protein-modified beads–either hCG target or Bovine Serum Albumin (it’s a common molecular biology blocking agent). After rinsing unbound aptamers from the beads, the remaining aptamers were assayed by the addition of HRP-conjugated streptavidin and the subsequent HRP-catalysed oxidation of TMB. Similar to the EMSA and qPCR results, ELONA-based screening of the aptamer candidates (Fig 4C) identified sequences R4_64 and R6_5 as capable of binding to hCG, as well as lower levels of R5_4 binding. The control sequence R4_1 exhibited low levels of binding to either protein, similar in extents to the “No DNA” control samples. Similar to qPCR results, none of the sequences exhibited significantly-different binding to BSA-modified beads, compared to the “No DNA” sample, indicating a lack of binding to this protein (Fig 4C). Relative to their BSA controls, all three tested aptamers (R4_64; R 5_4 and R6_5) demonstrated significant binding to hCG-modified beads (**†** annotations). Furthermore, sequence R6_5 exhibited a significantly higher colorimetric signal compared to the No DNA control sample (* annotations); higher (but not significantly-higher) responses were evident for R4_64 (Fig 4C).

An alternative binding approach was investigated using a low-cost paper surface on which the hCG protein target was immobilized onto cellulose, using EDC/NHS coupling (Fig 4D). As with the bead-based assay (Fig 4C), biotinylated, truncated, aptamer sequences retained after extensive washing were considered to be proportional to the colorimetric signal generated by TMB, producing a blue colour within the wells by the end of the assay (Fig 4D, upper panel). Using this approach, all sequences tested showed increased signal compared to the “No DNA” control, however, only sequence R4_64 generated significantly more colorimetric signal than the R4_1 sequence (* annotation in Fig 4D). These results, in agreement with the previous binding assays, indicate that R4_64 is able to recognise and bind to hCG.

Viewed overall (Fig 4A–4D), the comprehensive screening techniques employed indicated that the aptamer sequences identified from NGS analysis–R4_64, R5_4 and R6_5 –consistently bound to the hCG target. Overall, the consistency of binding the different sequences could be evaluated as significant/specific binding in the following order: sequence R4_64 (which exhibited significant/specific binding for all assays used here), followed by both sequences R6_5 and R5_4 (which each demonstrated assay-dependent binding). This highlights the NGS-based rational approach for selection of sequences based on enrichment during SELEX. They also emphasise the suitability of particular sequences to particular techniques and the need for investigating multiple methodologies when screening novel aptamers found by others e.g. [42, 43, 46, 47].

Correspondingly, the sequences identified by NGS analysis as unlikely to bind the target to a great degree showed expected results. The Lib_1 sequence demonstrated minimal binding when evaluated using EMSA (Fig 4A) and qPCR–this sequence showed the least amount of DNA bound to hCG-functionalised beads (Fig 4B). Similarly, the amplification artefact sequence R4_1, which showed very similar behaviour to Lib_1 through selection during NGS evaluation (Fig 3), displayed little evident binding to the target by the assay techniques used here (Figs 4A, 4C and 4D). Viewed together, these results suggest that this group of sequences were maintained in the SELEX pool as a consequence of their ready amplification during PCR, rather than their target binding specificity. By thoroughly examining the evolving selection pool through SELEX by NGS, from the very beginning by including the unselected library and the no-template control pool, these anomalous high frequency but low affinity sequences can be identified. This analysis can then exclude these parasite sequences from potential aptamer validation experiments, which can be costly and labour-intensive.

### 3.4 Identification of hCG aptamers specific to different epitopes of hCG

**Fig 5:**
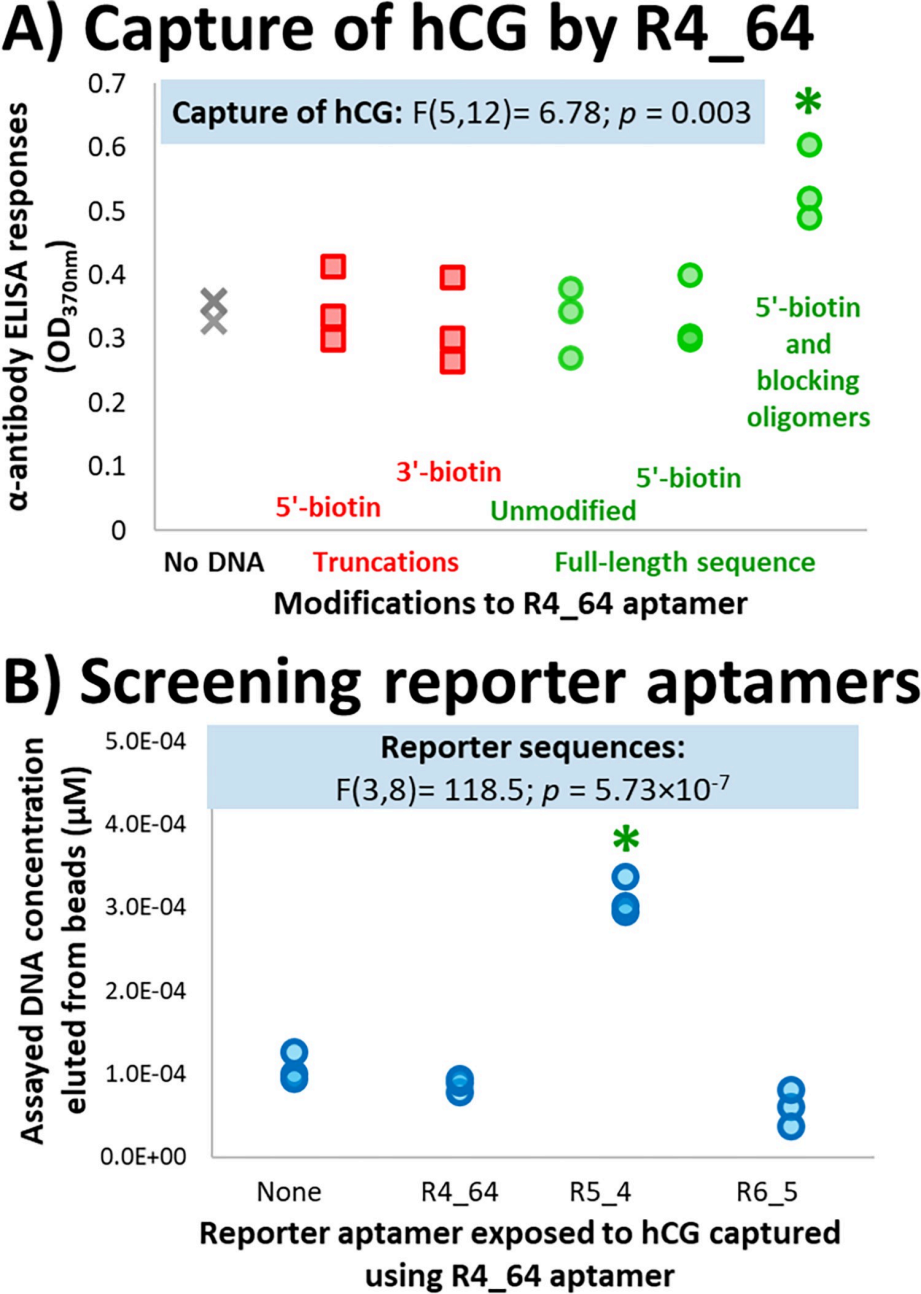
Competitive assays used to elucidate aptamer candidates′ target epitope specificity. **A) Evaluation of conditions required to capture hCG when using aptamer sequence R4_64.** Wells were coated with streptavidin, subsequently modified with biotinylated aptamer sequences and exposed to hCG. hCG captured by this system was quantified using the anti-α subunit antibody, subsequently using a HRP-conjugated secondary antibody and TMB to quantify the presence of the anti-α antibody. Different aptamer conditions were compared to control sequences which lacked any biotin modification (unmodified full-length R4_64) and any capturing DNA (no DNA). Triplicate measurements were generated and shown. Annotations: *—Sequences exhibiting significantly higher binding to hCG-modified beads compared to “No DNA” control samples (one-way ANOVA results annotated in Fig; sequences identified using subsequent Tukey HSD *post-hoc* test, *p* ≤ 0.05). **B) Screening of aptamer sequences capable of binding to hCG sites that do not compete with R4_64′s binding.** Full-length R4_64, folded in the presence of the complementary blocking oligonucleotides, was immobilised onto streptavidin-coated magnetic beads through its attached biotin moiety. Beads were exposed to hCG and subsequently, full-length, oligomer-blocked aptamer candidates. Following DNA elution, aptamers bound to the beads were quantified by SYBR qPCR. Triplicate measurements were made and shown. Annotations: *—Sequences exhibiting significantly higher binding to hCG captured by R4_64, compared to the “None” control samples (one-way ANOVA results annotated in Fig; sequences identified using subsequent Tukey HSD *post-hoc* test, *p* ≤ 0.05).

To determine whether the targeted selection strategy employed here allowed for the successful identification of aptamers specific to different regions of the protein target, competitive molecular biology assays were used (Fig 5). Exploiting the known binding sites of the available commercial antibodies allowed competition assays to be used where aptamer sequences sharing these sites would be eliminated and therefore reduce the colorimetric signals reported by the SA-HRP detection system as less biotinylated aptamer would be present (Fig 5A).

On the basis of consistency of binding (Fig 4), R4_64 was selected for a further study, evaluating its ability to capture the target, via a bead-based ELISA assay (Fig 5A). Variations of the same sequence (both full-length sequence and truncations of the variable region of the aptamer and 5′ and 3′ biotin modifications) were immobilised through their biotin modifications onto streptavidin-coated beads, which were exposed to hCG. Bound hCG was subsequently detected using an antibody specific to the α-subunit of hCG.

Only when the full-length sequence that was folded in the presence of the complementary oligonucleotides was used, was a detectable and statistically-significant colorimetric signal produced (* annotation, Fig 5A) when the concentration of hCG’s α subunit was assayed using its corresponding antibody. This oligonucleotide was therefore able to capture and immobilise free hCG from the solution, but only when the aptamer was prepared in a configuration similar to the conditions experienced during SELEX. These results suggest that the correct folding of this aptamer depends on the primer binding sites being blocked with oligomers, presumably limiting nucleotide interactions of these regions with the internal region selected by SELEX. The attachment of the biotin reporter at the end of this inert double-stranded region may also prevent steric hindrance when the DNA molecule is immobilised onto the magnetic bead via streptavidin. It has previously been shown that including a spacer region between immobilised aptamers and solid surfaces improves folding and minimises steric hindrance [47]; the optimal spacer configuration significantly affecting the immobilised aptamers′ target binding ability [48] and is supported by the data presented here.

Having demonstrated the ability to capture hCG, oligomer-blocked non-biotinylated R4_64 was used as a potential competitor, to investigate whether any of the other aptamer candidates could be combined with it to detect hCG, equivalent to an antibody sandwich assay. Beads containing hCG captured by R4_64 were exposed to full-length reporter aptamer sequences that were also folded in the presence of the blocking oligonucleotides. The DNA on the beads (assumed to be a combination of the R4_64 capture aptamer and the screened reporter aptamers) was eluted and quantified by qPCR (Fig 5B). As expected, the use of folded R4_64 aptamer as a reporting agent exhibited no increase in the DNA yielded from the beads, indicating that the site exploited by the R4_64 during hCG capture is occupied. The similar DNA yield from the use of R6_5 also indicated a competition in binding sites between R4_64 and R6_5, indicating that both potentially bind at or near the β_1_ epitope of hCG.

Competition assay results (Fig 5B) suggest that R4_64 and R6_5 aptamer candidates have the same, or overlapping binding sites on the hCG target molecule, possibly the β_1_-epitope. R5_4 does not share this specificity, but rather appears to recognise a distant and distinct site on the β-subunit and can be used in combination with R4_64, and possibly also R6_5. Competition between R4_64 and R6_5 was initially unexpected, since specificity of binding to the β1 epitope formed a selective pressure from Round 6 of SELEX, onwards. It was expected that DNA sequences originating from Rounds 6–9 would be enriched specifically against the β_1_ epitope–i.e. in these studies, sequence R6_5. Although R4_64 originated earlier in selection, before epitope specific selection was employed, the β_1_-epitope was exposed and available for aptamer binding during all rounds of selection. This epitope comprises only approximately 4.5% of the surface area of the β subunit of hCG, but any sequences binding to this region of the protein would be expected to increase in abundance throughout SELEX, even after the antibody-blocked hCG formed part of the negative selection step was introduced. The enrichment of aptamers binding to β_1_ in SELEX is supported by the studies monitoring the DNA eluted from beads at each selection round (Fig 2A): an increase in the DNA pool recovered from the positive selection beads is observed at round 6, where the selection strategy changed to include β_1_-epitope specificity. This suggests that the previous five rounds of selection had already started to condition the pool towards this region of the protein target.

To study the competition observed between R4_64 and R6_5 in Fig 5, putative tertiary structures of the variable regions of the sequences reported in this study were generated using *in silico* methods [49–54] (S4.1 contains detailed methodology in S4 File) and compared to the crystallographic structure of the subunit of hCG (1HRP.PDB [6]) (S4.2 Table in S4 File). The variable regions of the *in-silico* folded aptamers are of similar size to the β subunit of hCG: they have similar volumes (12.6×10^3^ Å^3^ for hCG’s β subunit, compared to an average volume of 10.6 ± 0.3 Å^3^ for the predicted tertiary structures of sequences R4_1, R4_64, R5_4, and R6_5) and similar surface areas (6.5×10^3^ Å^2^, compared to 5.8 ± 0.2 ×10^3^ Å^2^ for the protein’s βsubunit and folded aptamers’ variable regions, respectively).

Given the above, it is likely that, when binding to the surface of the β subunit of hCG, aptamers generated in this study may conceivably cover much of the available binding surface, blocking it from further interactions. Given the small size of the β_1_ epitope (3/111 amino acids), its central location on the surface of hCG (S4.2 Fig in S4 File) and its relatively nonpolar composition [16], it is likely that many of the earlier aptamers enriched between Rounds 1–5 (i.e. 4_64 and 5_4) may include this epitope as part of their aptamer-target complex. Similarly, it is expected that aptamers enriched during Rounds 6 onwards will also incorporate other regions of hCG during complexation, in addition to β_1_.

Preliminary docking simulations of the folded variable regions using the shape complementation server GRAMM (S4.2 Fig in S4 File) reiterate that large stretches of hCG might participate in binding with the aptamers generated in this study. Many of the predicted aptamer-target structures generated during docking indicated that aptamers might bind to sites of hCG that encompass the β_1_ epitope (R4_64, R6_5 and R4_1), or at regions proximal to β_1_ (R5_4). The low degree of overlap between R4_64 and R5_4 when the most-favourable predicted binding sites are overlaid might provide some evidence for the lack of competition between the two sequences that was experimentally-demonstrated (Fig 5B). Given the ongoing challenges and complexities in using entirely *in silico* methods to predict tertiary structures based on sequences alone [54, 55] and modelling the subsequent binding behaviour of DNA aptamers compared to *in vitro* binding studies [54, 55], these docking results are considered to be preliminary and will require additional confirmatory studies, such as crystallography, to elucidate binding conformation and epitopes participating in binding.

## 4. Conclusions

This study describes a novel predetermined epitope-targeted selection strategy which was used to identify aptamers recognising two distinct sites of the hCG molecule—the β subunit and the β_1_-epitope conserved amongst hCG variants. This was achieved by iteratively exposing the DNA pool to magnetic beads functionalised with either target molecules (positive selection) or non-target molecules (negative and counter selection). While previous SELEX strategies have been described to isolate aptamers against a particular region of a target, none reported have used antibody-blocked target molecules as a negative selection pressure during SELEX to direct binding to a specific epitope, as was used here. This strategy offers a number of advantages over other methods, including the need for only a single SELEX experiment to enrich different pools of aptamers capable of binding discrete sites on the target. This strategy might prove useful for similar studies interested in generating aptamer pairs for use in combination in sandwiching assays, similar to antibody-based sensor formats. Additionally, the change of selection pressure within the selection process, analysed by NGS, allowed for discrimination of suspected amplifier artefact sequences and genuine target binding sequences.

A variety of qualitative binding assays to evaluate binding were conducted; overall, these indicated that sequences R4_64 and R6_5 show relatively consistent binding to the target across the tested methods, highlighting their potential as possible biorecognition agents in biosensors. Competition assays revealed that R4_64 and R6_5 bound to an overlapping site on the target, likely the β_1_-epitope, which was not shared by R5_4. Competition between these aptamers for binding space on the target is a limitation of the aptamers generated in this study, due to the relative sizes of the target and the folded aptamers, as indicated by preliminary *in silico* analysis. This may preclude many of the sequences identified during NGS from being immediately applicable in sandwich formats for assays; additional competition screening assays such as those conducted in this study will need to be conducted to find compatible binding aptamer pairs. Experimentally, the preliminary binding sites specificity of R4_64 and R5_4 appear to allow these aptamers to simultaneously capture and report on the target, indicating potential application for future use in a sandwich format assay.

These sequences therefore display the potential to be used in subsequent applications as hCG-specific biorecognition agents, with further characterisation and optimisation to achieve this aim currently ongoing. The use of these aptamers in a sandwich-style diagnostic test might ensure recognition of the core β_1_ epitope and enable its detection in urine samples, in order to more accurately quantify the total hCG present in samples. Additional studies, including comparing binding affinities of the full-length and truncated regions and conducting more detailed analyses of the reported sequences by generating point-mutations of each sequence to identify essential residues for target binding are also ongoing.

## Supporting information

S1 File Specifics of SELEX strategy used to select site-specific hCG aptamers. (DOCX)

S2 File Comparison of numbers of NGS sequence reads obtained and analysed using the AptaSUITE bioinformatic platform. (DOCX)

S3 File Detailed methodology for the screening of aptamer candidates. (DOCX)

S4 File Preliminary *in silico* analysis of binding sites of aptamers and other sequences generated during SELEX. (DOCX)

S1 Raw images Raw, uncropped, gel images used for EMSA results presented in Fig 4A of the manuscript. (PDF)
