# Supplementary material for: Generation of epitope-specific hCG aptamers through a novel targeted selection approach
Source: PLoS One. 2024 Feb 23;19(2):e0295673. doi: 10.1371/journal.pone.0295673 (PMC10890750; doi:10.1371/journal.pone.0295673)
Supplement: S1 File — (DOCX) [file pone.0295673.s001.docx]

## S1: Specifics of SELEX strategy used to select site-specific hCG aptamers.

Table S1: SELEX strategy used to select site-specific hCG aptamers.

| SELEX round | Library DNA (pmol) | Negative selection | | | | Positive selection | | | | Wash regimen of beads before DNA elution. |
| --- | --- | --- | --- | --- | --- | --- | --- | --- | --- | --- |
|  |  | Bead coating | Protein (μg) | Beads (mg) | Incubation time (min) | Bead coating | Protein (μg) | Beads (mg) | Incubation time (min) |  |
| Stage 1: Aptamer pools enriched towards hCG protein | | | | | | | | | | |
| 1***** | 1000 | Tris | 0 | 5 | 60 | hCG | 70 | 5 | 60 | 3 x artificial urine |
| 2 | 200 | Tris | 0 | 1 | 60 | hCG | 14 | 1 | 60 | 5 × artificial urine with 0.005% ^v^/_v_ TWEEN 20 |
| 3 | 200 | Tris | 0 | 1 | 60 | FSH S_N_ | 14 | 1 | 60 | 5 × artificial urine with 0.005% ^v^/_v_ TWEEN 20 |
| 4***** | 200 | Tris | 0 | 1 | 60 | hCG | 10 | 1 | 60 | 5 × artificial urine with 0.005% ^v^/_v_ TWEEN 20 |
| 5***** | 20 | Tris | 0 | 1 | 60 | hCG | 10 | 1 | 60 | 5 × artificial urine with 0.005% ^v^/_v_ TWEEN 20 |
| Stage 2: Aptamer pools enriched towards β_1_ epitope of the hCG protein | | | | | | | | | | |
| 6***** | 20 | Ab- hCG | 10 Ab +  10 hCG | 1 | 75 | hCG | 10 | 1 | 45 | 6 × artificial urine with 0.005% ^v^/_v_ TWEEN 20 |
| 7 | 20 | Ab- hCG | 10 Ab +  10 hCG | 1 | 75 | FSH S_N_ | 10 | 1 | 45 | 6 × artificial urine with 0.005% ^v^/_v_ TWEEN 20 |
| 8***** | 20 | Ab- hCG | 10 Ab +  10 hCG | 1 | 75 | hCG | 10 | 1 | 45 | 6 × artificial urine with 0.005% ^v^/_v_ TWEEN 20 and 4 M NaCl |
| 9***** | 20 | Ab- hCG | 10 Ab +  10 hCG | 1 | 75 | hCG | 10 | 1 | 45 | 6 × artificial urine with 0.005% ^v^/_v_ TWEEN 20 and 4 M NaCl |

* - selection rounds analysed by Next-Generation Sequencing in this study.

Fig S1. Bead surface characterisation by ELISA style assay at each round of selection, by detecting the ɑ-subunit shared by hCG and FSH. The surface of the beads for both positive and negative selection steps, at every round of SELEX, was analysed using an HRP-conjugated rabbit anti-goat secondary antibody to the goat anti-hCG ɑ-subunit specific antibody capable of recognising hCG (in all positive selection rounds), FSH (in counter selection rounds 3 and 7) and β1-specific antibody coupled hCG (in the negative selection in rounds 6 - 9).

100 µg of beads for the first selection round, or 20 µg for each subsequent round, were tested in triplicate for each condition. TMB colour change was measured kinetically over twenty minutes and the rate of change is reported here. Error bars show standard deviation for triplicate values.

Conditions generating TMB signals statistically different to those of round 2 are shown as * for the negative selection steps and † for the positive selection steps.

To account for any variance in target loading during selection, especially considering the change in selection pressure used in this SELEX strategy, aliquots of the protein-bound beads used in SELEX were analysed using an α-subunit antibody based ELISA method to detect both hCG and FSH coupled to the magnetic beads (Fig S1). With the exception of the first selection round – which used five times more beads than any of the other rounds (Table S1) – no difference in the amount of target protein present on the magnetic beads for the positive selection samples (Tukey *post-hoc* test, Fig S1).

The consistency of TMB signal produced between positive selection rounds indicates that while some batch-to-batch variation does occur between positive selection bead sample for each round, this difference is not statistically significant (Fig S2). This, in turn, indicates that equivalent protein amounts were present throughout selection, even between the first (Rounds 1 - 5) and second phases (Rounds 6 - 9).
