# Supplementary material for: Generation of epitope-specific hCG aptamers through a novel targeted selection approach
Source: PLoS One. 2024 Feb 23;19(2):e0295673. doi: 10.1371/journal.pone.0295673 (PMC10890750; doi:10.1371/journal.pone.0295673)
Supplement: S2 File — (DOCX) [file pone.0295673.s002.docx]

## S2: Comparison of numbers of NGS sequence reads obtained and analysed using the AptaSUITE bioinformatic platform.

Table S2: Comparison of numbers of NGS sequence reads obtained and analysed using the AptaSUITE bioinformatic platform.

| **Sequence pool** | **Initial reads** | **Merged, trimmed and QC filtered** | **Unique sequences, percentage of pools** | **Top 15 000  n=2 clusters abundance in CPM** | **Enriched clusters found initially in pool  (% of top 15,00 clusters)** | |
| --- | --- | --- | --- | --- | --- | --- |
| **Control sample pools** | | | | | | |
| Initial Library | 1 136 316 | 1 076 687 | 98.79 | 67 763 | 57 007 | (84.3%) |
| No-template control | 703 954 | 685 382 | 50.85 | 228 608 | 228 608 | (100%) |
| **SELEX sample pools – first phase** | | | | | | |
| Round 1 | 1 867 344 | 1 770 915 | 99.14 | 17 027 | 3.3 | (0.02%) |
| Round 4 | 1 263 374 | 1 196 968 | 94.81 | 29 444 | 322 | (1.09%) |
| Round 5 | 1 682 308 | 1590013 | 83.71 | 40 848 | 703 | (1.72%) |
| **SELEX sample pools – second phase** | | | | | | |
| Round 6 | 1 532 961 | 1443487 | 69.37 | 63 300 | 52 857 | (83%) |
| Round 8 | 1 439 972 | 1360272 | 46.10 | 318 890 | 98 870 | (31.0%) |
| Round 9 | 1 172 576 | 1100523 | 51.76 | 412 940 | 57 386 | (13.9%) |

Multiple rounds of selection were sequenced by NGS and processed using AptaSUITE. The total number of raw reads initially obtained is shown for each sequenced round, as well as the numbers of filtered sequences and unique sequences obtained through analysis. Percentages of the filtered sequences for unique reads are shown. The enrichment of the pool was calculated as a decrease in the proportion of unique reads in the samples and the decrease in unique cluster proportions found as SELEX progressed.
