# Supplementary material for: Generation of epitope-specific hCG aptamers through a novel targeted selection approach
Source: PLoS One. 2024 Feb 23;19(2):e0295673. doi: 10.1371/journal.pone.0295673 (PMC10890750; doi:10.1371/journal.pone.0295673)
Supplement: S3 File — (DOCX) [file pone.0295673.s003.docx]

## S3: Detailed methodology for the screening of aptamer candidates

## S3.1 Screening of aptamer candidates by electrophoretic mobility shift assays (EMSAs)

Aptamer candidates were diluted to 250 nM in artificial urine and heat-treated by heating to 95 °C for ten minutes and gradually cooled to 21 ± 2 °C. A 27 pmol sample of hCG, dissolved in 0.15 M HEPES pH 7.0 was then added to 2.5 pmol of the aptamer and incubated at 21 ± 2 °C for one hour. The entire sample was combined with 2 µl of 6× loading dye and electrophoresed in 10% ^v^/_v_ PAGE gels at 80 V for 80 minutes. DNA bands were visualised after 20 minutes of 1×GelRed staining, using a BioRad Gel Doc™ EZ Imager. Each gel contained an hCG sample without the addition of any DNA was as a negative control, as well as a 2.5 pmol folded aptamer sample lacking hCG as each sequence’s standard. The pixel volume intensity of each aptamer band for the samples containing and lacking hCG were quantified using ImageJ software. Changes to the volume intensity of the main band associated with the aptamer in the presence of hCG was used to evaluate potential aptamer binding to hCG.

## S3.2 Screening of aptamer candidates by magnetic bead-based ELONA

Protein-modified magnetic beads were prepared by functionalising 1 mg of epoxy beads with 10 μg of either hCG or negative control proteins, BSA or HSA, as detailed in Section 2.2.1. Once functionalised, 72 µg aliquots of the beads were added to wells of a 96-well plate, which were pre-blocked with 3% ^w^/_v_ milk powder (in HMCKN-T buffer composed of 20 mM HEPES, 2 mM MgCl_2_, 2 mM CaCl_2_, 2mM KCl, 150 mM NaCl, pH 7.4 and 0.002% ^v^/_v_ TWEEN®-20). Wells containing bead samples were prepared in triplicate.

After two HMCKN-T washes of the beads, 100 µl of 250 nM of folded, biotin-modified, aptamer solution in artificial urine was added (prepared and heat-treated as detailed in Section S3.1) to each well. The aptamers were incubated with beads for one hour at 21 ± 2 °C; subsequently, each well was washed three times with 200 μl of HMCKN-T. The biotin on retained aptamers was detected by adding SA-HRP (KPL) – diluted 1 : 5 000 in HMCKN-T – to the wells, incubating at 21 ± 2 °C for 30 minutes. Following a further triple HMCN-T wash of the wells, SA-HRP was detected by adding 50 μl of 1-step Ultra TMB-ELISA substrate (ThermoScientific) to each well, allowing TMB oxidation to proceed at 21 ± 2 °C, in the dark, for 30 minutes. Thereafter, absorbance was measured at 370 nm and 650 nm, according to the manufacturer′s instructions. Each microtitre plate included DNA-free controls to account for non-specific interaction of reporting agents with the protein-modified beads, and to accommodate signal variation between plates due to minor variations in HRP loading and TMB incubation times.

## S3.3 Screening of aptamer candidates by magnetic bead-based capture ELONA

To test aptamer candidates′ ability to capture unimmobilised hCG, streptavidin-coated beads were prepared using 10 μg of streptavidin (Sigma) per mg magnetic beads. ELONA plates were prepared as described above using 60 μg of functionalised beads per well.

Folded, biotinylated 4_64 sequences (prepared as before in Sections S3.1) were immobilised onto the streptavidin-functionalised beads for 30 minutes at 21 ± 2 °C. Several variations of R4_64 were trialled: 5′-bioinylated truncated and 3′-bioinylated truncated, 5′-bioinylated-full-length and 5′-bioinylated full-length with blocking oligonucleotides sequences. Aptamer-labelled beads were washed three times with HMCKN-T and incubated with 100 μl of 1 μg/ml hCG in HMCKN-T for an hour at 21 ± 2 °C. hCG captured by aptamer-modified beads was detected adding 100 µl of a 1 : 5 000 dilution of the α-subunit specific primary antibody in HMCKN-T and incubating for 30 minutes at 21 ± 2 °C. Following three HMCKN-T washes, a 1 : 5 000 dilution of the HRP-conjugated secondary antibody was added and allowed to bind for 30 minutes at 21 ± 2 °C. Following three HMCKN-T washes, colorimetric TMB oxidation was developed as described above. DNA-free and unbiotinylated DNA control tests were included as negative controls.

## S3.4 Paper-format ELONA studies

Whatman chromatography paper (Grade 1, GE Healthcare Life Sciences) was used to create printed wells. The printed wells were 5 mm in diameter and surrounded by a 2.12 mm-wide hydrophobic wax barrier, printed onto the paper surface with a Xerox Colorqube 8870 printer. Before use, the wax was melted into the paper by heating to 100 °C for a few minutes, creating a hydrophobic barrier to contain the reaction within the wells during testing.

hCG was immobilised onto the surface of each well using EDC/NHS coupling. 0.1 M NHS and 0.4 M EDC stocks were prepared fresh in 0.15 M HEPES, pH 5.8. A 5 μl spot of freshly-mixed 1 : 1 EDC/NHS solution was applied to the centre of each wax-printed well and allowed to react for 15 minutes at 21 ± 2 °C. This was repeated with another 5 μl EDC/NHS aliquot. Each well was individually washed, three times, with 5 μl HEPES buffer, wicking excess fluid onto fresh paper toweling placed underneath the paper. 5 μl of 20 μg/ml hCG solution, diluting the stock solution in HEPES, was added to the paper wells and incubated at 21 ± 2 °C, in a humidified chamber, for one hour. Each well was subsequently washed three times with HEPES buffer. 2 µl of 2 μM dilutions of heat-treated, biotinylated aptamer sequences, in artificial urine, were added to each well and incubated at 21 ± 2 °C for two hours. Each well was subsequently individually washed, three times, with artificial urine.

The entire sheet of wax-printed paper was then submerged in 3% ^w^/_v_ milk powder in PBS-T and incubated under rocking for an hour at 21 ± 2 °C, to block the paper surface from non-specific binding. The entire sheet was washed three times, by submerging it into HEPES buffer. Biotinylated aptamers were detected using a polyclonal goat anti-biotin antibody (Abcam). The HRP-conjugated antibody was diluted 1:10 000 times in the milk powder block and 5 μl was added to the centre of each paper well. After 30 minutes’ incubation, the entire sheet was submerged in PBS-T for three washes and briefly air-dried on paper towelling. 5 μl of TMB substrate (SeraCare) was added per well and an image of the colour development was immediately captured using a desktop scanner (Cannon CanoScan LiDE 110). Quadruplicate wells used for each sample.

The pixel intensity of the interior of each well was calculated using ImageJ. Briefly, an RGB image file was split into the three colour channels and inverted. Each well was individually selected and added to the OI manager in ImageJ. The intensity of each spot, in each colour channel, was then measured and these values imported into a spreadsheet for analysis. The average value of the blank well (“No DNA” control) in each colour was subtracted from every other well and squared. The change in intensity (ΔI) for each well was calculated from the square root of the sum of the squares of RGB channels, as previously described [47].

## S3.4 qPCR detection of full-length aptamers on hCG coated beads

Magnetic beads, coated either with hCG or HSA, were prepared as outlined in Section 2.2.1. Aliquots of 50 μg of beads were added to the wells of a microtitre plate and washed twice with HMCKN-T. A 100 μl aliquot of 250 nM folded, unlabelled, full-length aptamer sequences were added and allowed to binding at 21 ± 2 °C for an hour, after which each well was washed three times with HMCKN-T and once with HMCKN, before being resuspended in 50 μl HMCKN. 1 μl of this suspension was used as the DNA template in a SYBR green real-time PCR amplification, as described Section 2.2.2.

To test whether aptamer sequences could be combined in a sandwich format, streptavidin coated beads were prepared and 25 μl of the 2 mg/ml bead suspension was added per well of a 96-well plate. 250 nM biotinylated full-length R4_64 DNA was folded in the presence of each complementary blocking oligonucleotide, in artificial urine, and 100 μl added per well and allowed to bind at 21 ± 2 °C for an hour. After three HMCKN-T washes, 100 μl of 1 μg/ml hCG was added per well and incubated at 21 ± 2 °C for one hour. Each well was washed three times with HMCKN-T, once with HMCKN and resuspended in 50 μl HMCKN. 1 μl of this bead resuspension was used as the template in a qPCR assay to quantify the amount of unbiotinylated DNA bound to the captured hCG target.
