# Supplementary material for: Generation of epitope-specific hCG aptamers through a novel targeted selection approach
Source: PLoS One. 2024 Feb 23;19(2):e0295673. doi: 10.1371/journal.pone.0295673 (PMC10890750; doi:10.1371/journal.pone.0295673)
Supplement: S4 File — (DOCX) [file pone.0295673.s004.docx]

**S4:** **Preliminary *in silico* analysis of binding sites of aptamers and other sequences generated during SELEX**

**S4.1 Methodology:**

To generate potential tertiary structures for these aptamers, a similar approach to that reported elsewhere [50] was used. Putative secondary structures of the variable regions were predicted using mfold (<http://www.unafold.org/mfold/applications/dna-folding-form.php>) [51], setting the salt content to 0.15 M and the folding temperature to 20 °C. RNA analogues were generated using the secondary structures using RNAComposer (https://rnacomposer.cs.put.poznan.pl/) [52], generating .PDB files of RNA molecules with possible tertiary structures, based on the predicted secondary structure. The RNA molecules within the .PDB files were manually converted to DNA in the following manner. Using Python Molecule Viewer v1.5.7 [53], the 2ʹ-OH group of the ribose sugar in all residues was deleted. The hydrogen bonded to C5 of the ring of uracil (5H) was replaced with a carbon atom using the ‘Change Element’ command in ChimeraX and converted to a methyl by adding hydrogens to the structure. Using a text editor, the residues were changed from their RNA designations (C,A,G,U) to their DNA designations (DC,DA,DG,DT) and the C1 atom of uracil’s nitrogenous group was renamed to C7 of thymine’s.

Aptamer-protein binding was preliminarily assessed on the basis of shape-complementation using GRAMM (<https://gramm.compbio.ku.edu/>) [54], similar to other aptamer-protein studies reported [55]. The top 10 structures identified using this method were downloaded and manually compared. Conformers i.e. groups of aptamers forming complexes with highly-similar binding orientations and positions on the protein were then manually determined: from the groups of conformers, the maximum number of intermolecular hydrogen bonds connecting the aptamer to the protein were determined and presented below. Volumes and surface areas of the individual molecules were estimated using the “Measure Volume and Surface Area” tool of ChimeraX.

**S4.2 Results:**

**Table S5: Computed volumes and areas of the docking participants**

| **Participant name** | **Volume (**×10^3^ Å^3^**)** | **Surface area (**×10^3^ **Å^2^)** |
| --- | --- | --- |
| hCG, β subunit | 12.61 | 4.996 |
| Sequence R4_1 | 10.39 | 5.797 |
| Sequence R4_64 | 10.43 | 6.066 |
| Sequence R5_4 | 10.84 | 5.539 |
| Sequence R6_5 | 10.84 | 5.784 |

**S5 Fig: Comparison of putative binding sites for the aptamers generated during the study, generated via *in silico* shape-complementation docking.**

A) For the hCG molecule, the different subunits are annotated, along with the space-filled orange region denoting the β_1_ epitope.

B) – E) For the aptamer candidates reported, an image of one of the most-frequent conformer found for each sequence is presented above, along with the maximum number of intermolecular hydrogen bonds for that conformer.

F) An overlay of R4_64 and R5_4, showing that the predicted binding sites of these aptamers do not overlap significantly.
