# Supplementary material for: Generation of epitope-specific hCG aptamers through a novel targeted selection approach
Source: PLoS One. 2024 Feb 23;19(2):e0295673. doi: 10.1371/journal.pone.0295673 (PMC10890750; doi:10.1371/journal.pone.0295673)
Supplement: S1 Raw images — (PDF) [file pone.0295673.s005.pdf]

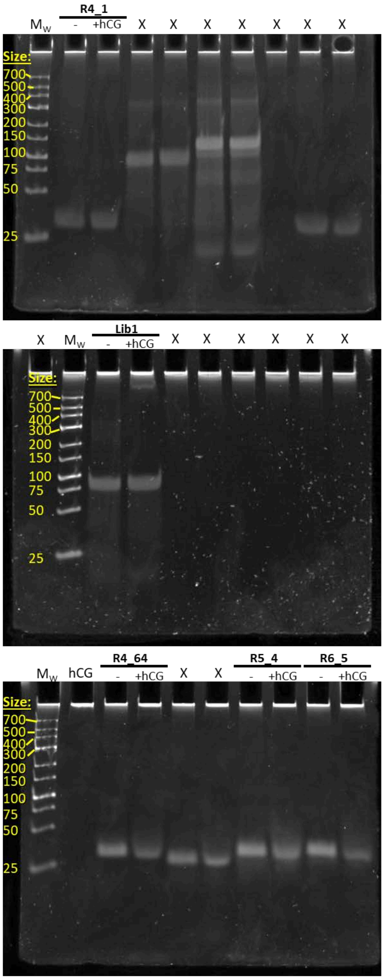

S5 Fig: Raw, uncropped, gel images used for EMSA results presented in Figure 4A of the manuscript. 2.5 pmol of each tested sequence was electrophoresed. **X** denotes either blank lanes or sequences not reported in the manuscript, - denotes sequences electropheresed without hCG and **+hCG** shows the same sequence electrophoresed with 27 pmol of hCG The bottom gel shows the electrophoretic profile of hCG without any aptamer candidate sequence.
